# Diffuse Axonal Injury and Oxidative Stress: A Comprehensive Review

**DOI:** 10.3390/ijms18122600

**Published:** 2017-12-02

**Authors:** Alessandro Frati, Daniela Cerretani, Anna Ida Fiaschi, Paola Frati, Vittorio Gatto, Raffaele La Russa, Alessandro Pesce, Enrica Pinchi, Alessandro Santurro, Flavia Fraschetti, Vittorio Fineschi

**Affiliations:** 1 Istituto di Ricovero e Cura a Carattere Scientifico (IRCCS) Neuromed, Via Atinense 18, 86077 Pozzilli, Italy; alessandro.frati@uniroma1.it (A.F.); paola.frati@fastwebnet.it (P.F.); raffaele.larussa@uniroma1.it (R.L.R.); 2 Department of Neurosciences, Mental Health, and Sensory Organs, Sant’Andrea Hospital, Sapienza University of Rome, Via di Grottarossa 1035, 00189 Rome, Italy; ale_pesce83@yahoo.it (A.P.); flavia.fraschetti@gmail.com (F.F.); 3 Department of Medicine, Surgery and Neuroscience, University of Siena, Viale Mario Bracci 16, 53100 Siena, Italy; daniela.cerretani@unisi.it (D.C.); annaida.fiaschi@unisi.it (A.I.F.); 4 Department of Anatomical, Histological, Forensic and Orthopaedic Sciences, Sapienza University of Rome, Viale Regina Elena 336, 00185 Rome, Italy; vittorio.gatto@uniroma1.it (V.G.); enrica.pinchi@uniroma1.it (E.P.); alessandro.santurro@uniroma1.it (A.S.)

**Keywords:** traumatic brain injury, oxidative stress, reactive oxygen species, immunohistochemistry, biomarkers

## Abstract

Traumatic brain injury (TBI) is one of the world’s leading causes of morbidity and mortality among young individuals. TBI applies powerful rotational and translational forces to the brain parenchyma, which results in a traumatic diffuse axonal injury (DAI) responsible for brain swelling and neuronal death. Following TBI, axonal degeneration has been identified as a progressive process that starts with disrupted axonal transport causing axonal swelling, followed by secondary axonal disconnection and Wallerian degeneration. These modifications in the axonal cytoskeleton interrupt the axoplasmic transport mechanisms, causing the gradual gathering of transport products so as to generate axonal swellings and modifications in neuronal homeostasis. Oxidative stress with consequent impairment of endogenous antioxidant defense mechanisms plays a significant role in the secondary events leading to neuronal death. Studies support the role of an altered axonal calcium homeostasis as a mechanism in the secondary damage of axon, and suggest that calcium channel blocker can alleviate the secondary damage, as well as other mechanisms implied in the secondary injury, and could be targeted as a candidate for therapeutic approaches. Reactive oxygen species (ROS)-mediated axonal degeneration is mainly caused by extracellular Ca^2+^. Increases in the defense mechanisms through the use of exogenous antioxidants may be neuroprotective, particularly if they are given within the neuroprotective time window. A promising potential therapeutic target for DAI is to directly address mitochondria-related injury or to modulate energetic axonal energy failure.

## 1. Introduction

Traumatic brain injury (TBI) can result in long-term damage in as many as 50% of affected individuals [1]. It is estimated that in 2020, TBI will become the third leading cause of permanent disability and mortality worldwide [2], with major economic implications for national health systems: in the United States alone, it is estimated that 1.7 million persons will suffer from TBI each year with an estimated expense of 60 billion dollars for specific medical treatments [3,4].

TBI is one of the most frequent causes of traumatic axonal damage, commonly known as diffuse axonal injury (DAI). While the first descriptions of experimental models of the diffuse morphological abnormalities of the white matter linked to shear and tensile forces on brain parenchyma were reported in the 1940s [5,6], Strich, in a classical paper from 1956 [7], was the first to fully recognize and describe the histopathological appearance of the white matter after severe traumatic brain injury. Adams [8,9] introduced the term “diffuse axonal injury”. He classified DAI in a three-step grading system, according to the extent of axonal damage.

The introduction of the high-field magnetic resonance imaging (MRI) [10] and the improvement of immunostaining techniques [11] shed light on the mechanisms underlying DAI. A deeper knowledge of the physiopathology as well as a deeper understanding of the risk factors associated with poor clinical outcomes have improved functional and neurological prognosis of patients suffering from DAI [1].

Yet DAI itself, as well as another condition belonging to the neurotrauma family, chronic traumatic encephalopathy (CTE) [12,13], still appears to be extremely heterogeneous and elusive clinicopathological entities per nature [11], and complete knowledge of these conditions seems some way off. Oxidative stress plays a critical role in the genesis of the dramatic clinical phenotypes of DAI that seem to be unavoidably linked to the effects of a plethora of post-traumatic molecular and cellular changes, for instance, the imbalance between the production and removal of ROS, the releasing and activation of pro-inflammatory cytokines, and modifications in calcium metabolism. The translational and clinical research on DAI is producing an outstanding amount of evidence in a surprisingly fast time.

Therefore, the aim of this literature review is to identify and encompass all the relevant, strongest and most recent evidence in the pathophysiological, diagnostic and clinical aspects of acute DAI, with a special focus on the translational aspect of the most recent findings into specific diagnostic and prognostic benchmarks, in order to outline and discuss the factors leading to a poor functional and neurological prognosis of patients suffering from DAI, with special attention on connecting the preclinical and clinical aspects, along with the diagnostic implications in the management of this condition.

## 2. Mechanism of Injury

DAI, as far as current evidence suggests, is thought to be one of the main causes of post-traumatic loss of consciousness in the absence of detectable intracranial lesions on computed tomography (CT) [8]. It represents the morphological correlate of a rotational acceleration-deceleration traumatic brain injury through strain or shearing forces [14,15]. During a traumatic brain injury the brain is subjected to a multitude of forces, such as rotational, tensile and compressive stresses; the inertia of the brain leads to a dissociation between its relative movement and the cervical column, thus, when a rapid movement of the head is generated during trauma without a significant physical impact, translational, rotational and/or angular acceleration can lead to blood vessels stretching or to axonal damage, mostly in the case of rotational acceleration [16]. In this case, the damage affects the planes between tissues of different density (the grey-white matter junction), and the rotational center of mass in the intracranial space (the rostral brain stem) [17].

### Experimental Models

Due to the structural complexity of the human brain it is difficult to replicate the entire spectrum of dynamic forces acting in human DAI pathophysiology. Several animal models have been developed, such as open-head injury models like fluid percussion (FP) [18] and controlled cortical impact (CCI) systems [19,20], in which the mechanical force is applied directly to the dura mater exposed by a craniotomy, and closed-head injury (CHI) models in which the damage is induced by direct impact (for example striking the intact skull with a piston or dropping a weight on the intact skull), non-impact (blast) or inertial loading (by rapid rotation of the head in various planes) [21]. The DAI mechanism is presumably similar to those investigated in the aforementioned injury models, although open head models are focused on the fine axonal effects of mechanical trauma.

A recent neurotrauma model system called CHIMERA (Closed-head impact model of engineered rotational acceleration) has been developed by Namjoshi et al. in order to integrate biochemical, behavioral and neuropathological analyses after delivery impacts of defined energy to a closed skull with unconstrained head motion after impact, making this model suitable to investigate the pathophysiology of TBI [22].

Although it has been experimentally shown that acceleration/deceleration forces are sufficient for producing injury, in the real world the direct impact almost always comes first [11]. While subdural hematomas occur more frequently in falls on a firm surface, DAI is seen in vehicle occupants where impact on deformable surfaces prolongs and reduces the rate of deceleration. In DAI, the force of the initial impact does not need to be strong enough to cause skull fractures, but it is still capable of producing a diffuse brain injury.

An essential factor in the development of shear strain is the direction of the head movement: coronal head movement is associated with more severe diffuse damage than sagittal head movement [15].

Summary: DAI represents the morphological correlate of a rotational acceleration-deceleration traumatic brain injury. Although several models have been proposed the complexity of in vivo condition are extremely difficult to reproduce.

## 3. Physiopathology

Traumatic injury causes dynamic deformation of the brain parenchyma; as a consequence, there is a risk of stretch and shear injuries of axons and blood vessels. Due to their viscoelastic nature, white matter axons are susceptible to damage by high strain rates produced during traumatic brain injury [23]. Following a TBI, axonal degeneration is identified as a progression from disruption in axonal transport leading to axonal swelling, followed by secondary disconnection and Wallerian degeneration. These modifications in the axonal cytoskeleton interrupt the axoplasmic transport mechanisms, causing the gradual gathering of transport products to generate axonal swellings and modifications in neuronal homeostasis. The initial impact of the brain causes focal perturbation in the axon, resulting in an alteration of the axonal transport and an accumulation of the β-amyloid precursor protein (β-APP), a transmembrane glycoprotein widely represented in the membranous structures of the central nervous system, which can be detected within 2 h after damage [24]. The accumulation of this precursor is common in other conditions [12].

DAI is not only caused by primary axotomy from mechanical forces, but also from secondary axotomy due to a progressive molecular and cellular cascade of pathologic changes within the axon after initial shear stress at the time of injury [25]. Some studies support the role of an altered axonal calcium homeostasis in the mechanism of the secondary damage to the axon, neuron and vessel [26], and suggest that the calcium channel blocker, nimodipine can alleviate the secondary damage, which as well as other mechanisms implied in the secondary injury, could be targeted as a candidate for therapeutic approaches [17,22].

The altered calcium homeostasis is the consequence of the release of glutamate and other excitatory amino acids, and of neuron mechanical perturbations that lead to mechanoporation of the cell membrane. Calcium is involved in the activation of caspases and calpains, that play a role in the initiation of necrosis and apoptosis. Furthermore, the generation of free radicals and the release of hydrolytic enzymes from lysosomes have a role in cytotoxic cascades [17].

The neuroinflammatory response also contributes to the mechanism of damage. In DAI experimental models the immune response of microglial cells in the central nervous system has been frequently investigated. Venkatesan et al. [27] used Galectin-3/Mac-2 as a marker of a subpopulation of activated microglia involved in myelin degradation, suggesting an important role in the pathogenesis of DAI. In a study by Oehmichen et al. [28] using an immunohistochemical double-labeling technique, β-APP and CD68 have been detected for axonal alteration and for microglia infiltration respectively. β-APP and CD68 co-localization in half of the patients 5– 15 days after injury demonstrates the presence of microglial infiltration in areas of axonal alteration.

Other studies have demonstrated the involvement of cytokines in neuroinflammation, such as IL-1α, IL-1β [29], IL-6 [30], Tumor Necrosis Factor (TNF)-α [31], and adhesion molecules such as ICAM-1 and chemokines (MCP-1) [32,33].

Summary: β-APP accumulation, calcium homeostasis dysregulation and neuroinflammatory responses linked to cytokine activation concur with the pathophysiology of neuronal death in TBI.

## 4. TBI and Oxidative Stress

DAI, as a type of TBI, is associated with cytoskeletal alterations, represented by swellings or varicosities along the axons and terminal bulbs [34,35,36]. These axonal alterations may be caused by mechanical disruption, and subsequent increasing intracellular Ca^2+^ influx by the breaches in the axolemma [37,38,39]. In this process transmembrane active transport by Ca^2+^ channels could also be implicated. Excess intracellular Ca^2+^ may be driven in the mitochondria, where reactive oxygen species (ROS) are generated, inducing oxidative stress into the axon [37]. Oxidative stress is an event caused by the imbalance between biochemical processes leading to the production of ROS and those responsible for the removal of ROS, known as the enzymatic and non-enzymatic antioxidant cellular defense systems. The excessive production of ROS due to excitotoxicity and depletion of the endogenous antioxidant system induces peroxidation of cellular and vascular structures, protein oxidation, and inhibition of the mitochondrial electron transport chain [40], causing oxidative cellular damage.

### 4.1. Role of Mitochondria and Calcium

Mitochondria are the main cellular consumers of oxygen and hold numerous redox enzymes capable of transferring single electrons to oxygen, generating ROS superoxide (O_2_^−^). Mitochondria also carry a large antioxidant defense system to detoxify the ROS produced by the reactions we are going to describe. The transfer of electrons to oxygen, generating superoxide, is more probable when these redox carriers are in great quantity charged with electrons and the potential energy for transfer is high, as reflected by a high mitochondrial membrane potential. ROS generation is reduced when available electrons are few and potential energy for the transfer is low. Non-enzymatic components of the system principally include tocopherol, coenzyme Q10 and glutathione (GSH). Enzymatic components include manganese superoxide dismutase catalase, glutathione peroxidase, glutathione reductase (GR) and others. The regeneration of GSH (through GR) depends on Nicotinamide Adenine Dinucleotide Phosphate (NADPH), which is derived from substrates or the membrane potential. So, like ROS generation, antioxidant defenses are dependent on the redox and energetic state of mitochondria. In intact mitochondria, a large antioxidant defense capacity balances ROS generation, and there is little ROS production. Mitochondrial damage with the decrease of antioxidant defense capacity is a precondition for ROS production. Once this occurs, a vicious cycle can result by which ROS can further damage mitochondria, causing more free-radical formation and loss or consumption of antioxidant capacity [41] with the generation of oxidative stress that causes damage to cellular structures. Studies over the last two decades suggest that free radical generation and oxidative damage plays a significant role in post-traumatic secondary injury after TBI [42], particularly to neuronal structures such as axons. After axonal damage, an increase in intracellular Ca^2+^ occurs, primarily derived from release of the intracellular pool and dysregulation of Ca^2+^ metabolism [43,44]. Increases in cytoplasmic Ca^2+^ determine mitochondrial Ca^2+^ sequestration, resulting in ROS generation and oxidative stress [37]. In the work of Johnson et al. the ionic derangement following axonal trauma has been hypothesized as playing a pivotal role in post-injury, in both axonal degeneration and the persistent dysfunction of otherwise intact axons [36]. In particular, it was thought that high amounts of intra-axonal Ca^2+^ play a central role in the secondary damage to axons following mechanical deformation [45,46,47,48,49]. Maxwell et al. established indirect proof of post-traumatic calcium influx into axons via changes in calcium-ATPase activity after optic nerve stretch injury [50,51,52].Also, utilizing an in vitro axon stretch model, explicit visual evidence of calcium entry into axons immediately following trauma has been demonstrated [53]. According to Büki et al., DAI is produced by focal perturbations of the axolemma, permitting calcium influx to trigger local intra-axonal cytoskeletal and mitochondrial modifications that generate cyto-c release and the activation of the caspase enzyme cascade in axons. It has been hypothesized that this mitochondrial damage creates local bioenergetic failure, causing axonal failure and disconnection [54]. Mitochondria appear to play a critical role in the secondary injury that occurs after TBI [55,56], and mitochondrial dysfunction has been shown to be involved in excitatory amino acid-induced neurotoxicity [57,58,59,60]. Increased Ca^2+^ concentration also causes an increased release of excitatory neurotransmitters [61], with resultant caspase activation [62,63] and distal axonal degeneration [64]. Axonal spheroid formations originate from axonal swellings [65] in axons undergoing oxidative stress [66] and have been associated with an increase in neuronal Ca^2+^, ROS production, impaired mitochondria and protease activation [65,67,68]. In particular, increase in neuronal Ca^2+^ [69] has a key role in neurodegeneration. In the work of Barsukova, an adult mouse model of was used to investigate the role of Ca^2+^ and ROS in the configuration of axonal spheroids and cytoskeletal changes. ROS-mediated axonal changes are mainly caused by extracellular Ca^2+^. Removal of extracellular Ca^2+^, rather than blockade of mitochondrial Ca^2+^ release, is an efficient strategy in lowering intracellular Ca^2+^ and inhibiting spheroid formation [66,70].

### 4.2. Role of Mitochondrial Membrane Permeability

Mitochondrial impairment has a relevant role in determining axonal alteration, by ROS production and mitochondrial permeability transition pore (mPTP) generation [71]. mPTP is an internal membrane protein, generated as a consequence of Ca^2+^ gathering, that permits mitochondrial influx and efflux [72,73]. Buki et al. used a rodent model in an experiment that explored the primary neurons of impact acceleration head injury. This underlined that associated with calpain activation, the related axonal cytoskeleton and organelles also demonstrated change consistent with calcium overloading as reflected in neurofilament sidearm modification and compaction, and local mitochondrial swelling with disruption of their cristae. They proposed that such mitochondrial perturbation was a terminal event in the local death of the axon [54]. It was recognized that mitochondrial swelling was fully coherent with a calcium induced opening of the mPTP and they supported this thesis through the use of cyclosporin-A, an inhibitor of the mPTP, which provides mitochondrial protection and cytoskeletal changes [74,75,76,77]. Cyclosporin A, a drug that binds and inhibits cyclophilin D, provided some interesting but not definitive results. Cyclophilin D is a protein complex involved in the modulation of mPTP77 in DAI models, inhibiting mPTP activation. Cyclosporin A failed to reduce axonal swelling in neurons exposed to ROS [66], but mitigates cytoskeletal and axonal alterations [78].

The role of intra- and extracellular Ca^2+^ in elevating axoplasmic concentration, in response to H_2_O_2_ exposition, has been studied by Barsukova in an adult mouse model. In this study, during exogenous oxidative stress the lack of extracellular calcium did not affect axoplasmic Ca^2+^ concentration, while generation of mPTP permitted Ca^2+^ mitochondrial release [69]. As a result, the lack of extracellular calcium associated with the application of cyclosporin A, completely abolished increases in axoplasmic Ca^2+^ [79]. These results demonstrate that extracellular Ca^2+^ and mPTP activation have a primary and a secondary role, respectively, in determining axonal alterations in response to ROS [66].

Inhibition of the formation of the mPTP has been described as a means prevent cytoskeletal changes and axonal degeneration subsequent to in vivo impact acceleration brain injury [54,75,76,80].

### 4.3. Oxidative Stress, Calcium Influx and Calpain Activation

**Figure 1:**
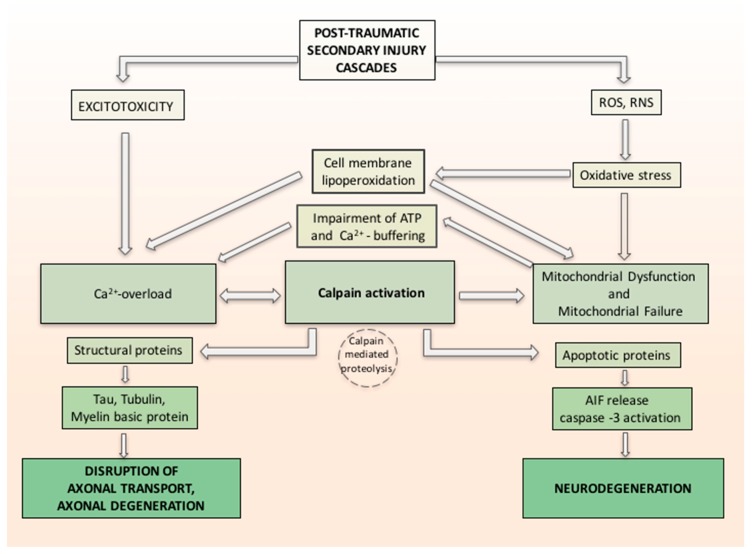
Hypothetical inter-relationship between (traumatic brain injury) TBI-induced oxidative damage and neurodegeneration. Secondary injury cascade in TBI induces oxidative stress related to increase of free radicals reactive oxygen species/reactive nitrogen species (ROS/RNS) and increase calcium entry both from intracellular stores and injury-induced increases in glutamate (excitotoxicity). Oxidative stress induces cell membrane lipoperoxidation and calcium release, which activates calpain. ROS and RNS induced oxidative damage in neuronal mitochondria and compromise Ca^2+^ homeostasis. Activated calpain mediates further Ca^2+^ entry, forming a positive feedback loop and induces mitochondrial membrane permeability and releases the apoptosis inducing factor (AIF) from mitochondria. Caspase-3 is also activated by Calpain-1. The released AIF and activated caspase-3 together induce neurodegeneration. Activated calpain proteolyzes large groups of cellular proteins varying from structural proteins and soluble proteins (e.g., apoptotic proteins). Changing either or both the structure or activity of the protein substrates can have important effects such as axonal deterioration and neuronal death.

In a study by Yamada et al. [81], the relation between oxidative stress, calcium influx and calpain-1 activation in primary neurons was investigated. The calpains are calcium-regulated cysteine proteases involved in ruling cell death pathways. Yamada demonstrated the probable molecular machinery of calpain-1 isoform in apoptosis and proved that it plays a practical role in the regulation of extracellular calcium influx and apoptosis in primary neurons exposed to oxidative stress. Oxidative stress causes calcium release, which activates calpain-1. Activated calpain-1 mediates further Ca^2+^ entry, generating a positive feedback loop. Then, activated calpain-1 induces mPTP and releases the apoptosis inducing factor (AIF) from mitochondria. Caspase-3 is also activated by Calpain-1. The released AIF and activated caspase-3 together induce DNA fragmentation and apoptosis [81]. Associated with calpain activation, the related axonal cytoskeleton and organelles also demonstrated alterations consistent with calcium overloading as reflected in neurofilament sidearm modification and compaction, microtubular loss, and local mitochondrial swelling with disruption of their cristae, with the hypothesis that such mitochondrial perturbation was a conclusive phenomenon in the local death of the axon [54]. Activated calpain proteolyzes large groups of cellular proteins, varying from structural proteins to membrane-bound proteins (receptors, channels) and soluble proteins (enzymes and apoptotic proteins), to nuclear transcription factors. Changing either or both the structure or activity of the protein substrates can have important effects, from regulating signal transduction to axonal deterioration and neuronal death (Figure 1) [82].

Summary: oxidative stress, caused by the post-traumatic imbalance between the ROS production and degradation plays a critical role in axonal and neuronal damage.

## 5. Pathological Anatomy and Morphologic Findings

### 5.1. Macroscopic Findings

The structural features of DAI were defined by Adams et al. [8,83] using a series of 45 fatal cases. In its most severe form, DAI has three distinctive structural features:

1. Diffuse supratentorial damage to axons (grade I)
2. A focal lesion in the corpus callosum (grade II)
3. A focal lesion or lesions in the rostral brain stem (grade III).

The focal lesions can often be identified macroscopically postmortem. They are usually hemorrhagic in patients with short survival, but they are difficult to identify at necropsy because they become shrunken scars; nevertheless, they are often brown in color because of the persistence of hemosiderin [8].

The lesion in the corpus callosum typically occurs in its inferior part and to one side of the midline. If it extends to the midline there is often disruption of the interventricular septum with an intraventricular hemorrhage.

The lesions of the rostral brain stem characteristically localize in the dorsolateral quadrant or quadrants adjacent to a superior cerebellar peduncle.

The axonal damage can be seen only on microscopical examination. In these cases, the diagnosis of DAI cannot be made without adequate histological studies [83,84].

If a patient with DAI survives for several months, the loss of bulk of the white matter causes a progressive enlargement of the ventricular system and at a postmortem examination it could be confused with post-traumatic hydrocephalus [8].

### 5.2. Microscopic Findings

The diagnosis of DAI must be confirmed by the microscopic finding of damaged axons: the swollen axonal varicosities and the axonal bulbs. The two main attributes in the pathological diagnosis of DAI are (1) the presence of diffuse/multifocal axonal damage in the white matter and (2) the fact that it is widespread in many brain regions, of which at least one should be located above and one below the tentorium [11].

The axonal damage takes three forms depending on the duration of survival. In their experimental studies on primates, Gennarelli et al. [15,83] graded axonal injury into three grades of severity:

- Grade 1: there is scattered axonal retraction balls in the parasagittal white matter of the cerebral hemispheres, the corpus callosum, the brain stem and, less commonly, the cerebellum
- Grade 2: in addition to axonal damage in the white matter of the cerebral hemisphere, there is a focal lesion in the corpus callosum
- Grade 3: in addition to axonal damage in the white matter of the hemispheres, the focal lesions are present in the dorsolateral quadrant of the rostral brain stem and the corpus callosum.

They concluded that the amount and distribution of axonal damage correlated well with the duration and severity of coma and the eventual outcome. On the basis of the time of survival, there are some characteristic features: the presence of a large number of axonal swellings in the white matter of the corpus callosum and the dorsolateral quadrant of the rostral brain stem as well as the white matter of cerebral hemispheres, cerebellum and brain stem, showed in patients with short-term survival rates (days); the small clusters of microglia throughout the white matter in patients of intermediate-term survival rates (weeks) ; the occurrence of Wallerian-type degeneration in the white matter throughout the cerebral hemispheres, the brain stem and the spinal cord, as a typical feature in patients who survive for many months [8].

Recently, Hill et al. pointed out that the term “retraction bulbs” is not exhaustive of the classical histological finding since the typical morphology is linked to the dysfunctional axonal transport rather than axonal retraction. Again, the previously defined “retraction ball” should be named “axonal bulb” to describe abnormal axonal profiles with a large single swelling [85]. Routinely, tinctorial stains, such as hematoxylin and eosin or silver impregnation techniques have been used to diagnose typical TBI features.

### 5.3. Immunohistochemistry and Stainings

**Figure 2:**
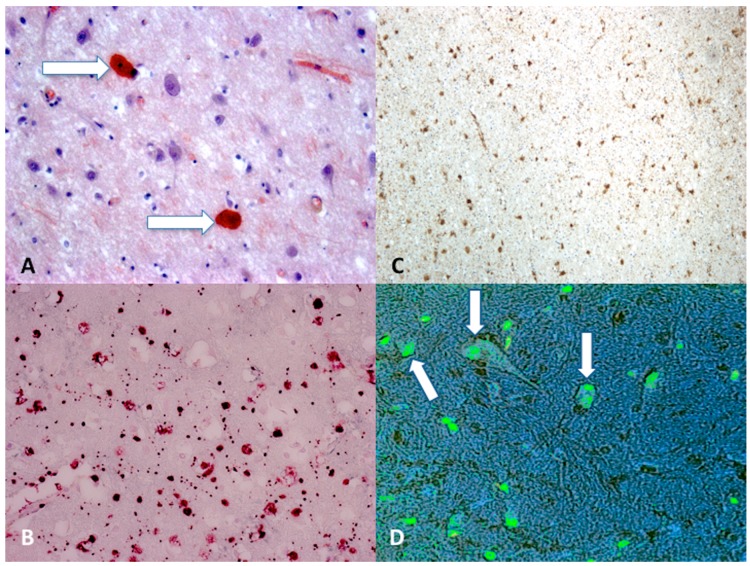
(**A**) Scattered axonal retraction balls stained with Congo Red (arrows), scale bar: ×250; (**B**) diffuse β-APP positivity expression in the corpus callosum is an indicator of axonal injury (grade II), scale bar: ×80; (**C**) β-APP (brown reactions) reaction exhibited a strong positive reaction typically occurring in the dorsolateral quadrant or quadrants adjacent to a superior cerebellar peduncle (grade III), scale bar: ×100; (**D**) morphological features of neuronal apoptosis (green) associated with marked condensation of chromatin and its fragmentation into discrete bodies (arrows), scale bar: ×250.

Today, the gold standard for immunohistochemistry in DAI cases is the search for β-APP (Figure 2). It has been demonstrated that finding damaged axons occurs within 12–24 h, so in cases with a short survival time it is difficult to detect DAI using conventional techniques, such as hematoxylin-eosin staining, which can identify the axon injury after about 24 h, and methods using impregnation with silver, which can reveal axon damage 12–18 h after the injury [83,86]. A immunohistochemical technique that uses antibodies against β-APP allows identification of damaged axons a 2–3 h after injury [87,88,89].

β-APP expression is an indicator of axonal injury, but it is not possible to distinguish between traumatic mechanisms injuries and damage due to ischemia/hypoxia. As a consequence, DAI cannot be assumed as a specific feature of mechanical injury alone.

Niess et al. [14] examined 450 non-selected human brains in order to estimate the overall incidence of DAI, assessing the axonal damage identifying β-APP in samples from pons and cerebrum. They stated that the biomechanical mechanisms in the trauma group are responsible for the presence of axonal damage mainly in the pons area, which is different to the more generalized pathomechanism in the intoxication group, which showed a much higher extent of axonal damage in both pons and cerebrum. Hayashi et al. [90] examined whether there are differences in the morphological pattern of axonal bulbs in trauma and hypoxia in sections of corpus callosum immunostained for β-APP. They found two different patterns. Similar investigations have been carried out by Oehmichen et al. [91] and Graham et al. [92] in order to find characteristic patterns and distributions of damaged axons that could indicate a traumatic origin of lesions.

### 5.4. Biomarkers

The processes characterizing DAI physiopathology determine the presence of potential biomarkers for monitoring specific processes, assessing the severity of the injury, and also developing new therapeutic strategies. A promising potential therapeutic target in DAI is direct to address mitochondria related injury or to modulate energetic axonal energy failure. As previously mentioned, caspase and calpain are calcium-dependent enzymes with a primary role in cytoskeletal alteration. Their activity causes the production of several proteolytic products, potentially considered as biomarkers of DAI, and extraordinarily important for the management of TBI (Table 1). In vivo, the inhibition of calpain and calcineurin is able to mitigate axonal degeneration. αII spectrin breakdown products (SBDP145, SBDP150, SBDP120) [93] derived from the cleavage of αII spectrin, a cytoskeleton component that stabilizes the nodal structure of myelinated axons. Some reports have demonstrated an elevation of αII spectrin and SBDPs after mechanical injury [94,95,96]. SBDPs increase within 6 h after injury and reach a peak in 2–3 days in cerebrospinal fluid (CSF). SBDPs have been found in other central nervous system injury of various origin [97], but they reach higher levels in TBI than in any other CNS disorder associated with raised ICP.

Another cytoskeletal injury marker is represented by neurofilament protein (NF), which undergoes proteolysis by caspase and calpain [98]. NF subunits have been found in the corpus callosum of TBI patients [96]. A recent study by Shibahashi et al. [99] investigated the usefulness of serum phosphorylated neurofilament heavy subunit (pNF-H) at 24 and 72 h after TBI, as a predictive marker for the outcome (vegetative state or death) of patients at 6 months. Conversely, Neurofilament Light chain (NF-L) protein serum level has been recently advocated as a potentially promising predictor of the 12 months neurological outcome of patients with an MRI demonstrated DAI [100]. This evidence, being inferred from relatively small samples of patients, deserves further confirmation by more extensive prospective trials.

Also, GFAP [101], microtubule-associated protein tau [102,103] and amyloid β peptide (Aβ42) [104] have been proposed as diagnostic and prognostic biomarkers in traumatic brain injury (TBI). Bogoslovsky et al. [105] recorded levels in plasma from the acute through subacute stages after TBI (24 h, 30 days and 90 days after the TBI). The levels were maximal at 24 h for GFAP and tau and at day 30 for Aβ42. GFAP, tau and Aβ42 increased up to 90 days after TBI compared with controls. A combination of all three biomarkers at 24 h and 30 days was found to be useful in both acute and subacute phases of TBI.

Others potential biomarkers of DAI are represented by S-100β protein and neuron specific enolase (NSE) [106]. S-100β is an acidic calcium binding protein [107] intensively investigated in severe head injury [108,109]. Increase of S-100β has been described in the early phase of injury in glial and Schwann cells, but S-100β cannot diffuse across the intact blood–brain barrier (BBB), so its serum concentrations depend on BBB permeability [110]. Conversely, recent evidence from others authors [111] focused attention on the contribution of an altered BBB, and did not confirm its role in influencing S-100β serum levels. Moreover, an elevation of S-100β in polytrauma could be a consequence of musculoskeletal trauma. Therefore, interpretation of S-100β should be considered with caution in polytrauma patients [112], notwithstanding that the effect of multi-trauma on S-100β has been found to be limited to the first 12 h [113]. In a study with ninety-two patients with severe TBI, GFAP was able to discriminate between severe disability and persistent vegetative state, despite both GFAP and S-100β being correlated with mortality [114]. A large, retrospective TBI outcome study [113] found that biomarkers S-100β and NSE are independently correlated to long-term functional outcome, but S-100β represents a more accurate outcome predictor and possibly a more clinically useful biomarker than NSE for TBI patients. Neuron specific enolase (NSE) is a glycolytic enzyme released into the extracellular space under pathological conditions during cell destruction. Levels are high in the CSF, but the serum concentration depends on the state of the BBB [115], and extracranial contribution is probably more problematic than S-100β. In pediatric TBI, NSE has been correlated with the Glasgow Outcome Score (GOS) [116].

Summary: β-Amyloid Precursor Protein (β-APP), NSE, Neurofilament Protein (NF), Glial Fibrillary Acidic Protein (GFAP) and S100 are critical biomarkers of neuronal death in TBI.

## 6. Diagnosis and Radiology

### 6.1. CT Scans

Unenhanced and Iodine contrast-enhanced brain CT scans are usually the first level of radiological examination for the severely head-injured patients [117]. Because of its easy access and rapid acquisition, a CT scan represents the imaging modality of choice for initial assessment of TBI and for the detection of acute hemorrhagic lesions for surgical intervention. It is especially useful for detecting intracranial traumatic pathologies in hemodynamically or neurologically unstable patients [118]. The Marshall classification shed the first light on the impact of CTs in the evaluation of the radiological severity of head trauma [117]. It was based on the observations of the Traumatic Coma Data Bank Study, and it described the condition of the basal cisterns, possible presence of midline shift, evacuated versus non-evacuated intracranial mass lesions and possible bony fragments. The CT scan has been improved [119] to show possible presence of intraventricular hemorrhage (IVH) and traumatic subarachnoid hemorrhage (tSAH), in order to provide a four-feature tool (midline shift, basal cisterns, IVH and tSAH) to predict post-traumatic morbidity and mortality. Computed tomography imaging does *not* definitely provide precise clues for the assessment of DAI or disclose the possible presence of DAI itself. However, it is a first-line examination for patients entering an Emergency Room, affected by severe TBI. The importance of the evaluation of IVH lies in the demonstrated association of its presence and severity as seen on the initial CT with DAI lesions and their severity as shown on the subsequent MRI, especially in the corpus callosum [120,121]. The correlation may be due to the shared shearing strain mechanism of subependymal vessels and mid-line brain structures’ axon damage. This information from the initial CT could be useful for selecting patients who should further undergo an MRI. Even if CT is highly sensitive in case of concurrent traumatic lacerations and other primary intracranial traumatic lesions, it is known that less than 20% of DAI patients present with macroscopically detectable “Stirch” hemorrhages; this methodology has the major limitation of underestimating the parenchymal effect of the head trauma and poorly discerns the diffuse axonal injury, so MRI is necessary to detect lesions that may be correlated to clinical symptoms, and that the initial CT is not able to explain [122]. Further investigations have produced different CT scan scoring systems to finely evaluate the first CT scan after a TBI [123]. Among such systems, the Rotterdam, Stockholm and Helsinki deserve a specific mention: the Rotterdam scoring system has gained popularity following its validation by the IMPACT trial—basically, it reweights the components of the Marshall system, evaluating such components by means of an arithmetic sum of single ordinal subscales; the Stockholm system is focused on the presence/absence of tSAH, IVH and possible intracranial traumatic blood collection in the same way as the Helsinki CT score.

### 6.2. MRI: Short-Term MRI and Follow-Up

Despite the limitations of applying MRI during the first weeks post-injury in patients who are unstable or do not cooperate, it is a more sensitive method than CT for detecting DAI, confirming the presence of small hemorrhagic lesions in the white matter of cerebral hemispheres, corpus callosum and brainstem [124,125]. Indeed, traumatic microbleeds in the white matter are considered radiological markers for DAI [126]. The use of MRI sequences sensitive to hemorrhages, such as susceptibility-weighted imaging (SWI) and T2*-weighted gradient echo (T2*GRE) can confirm DAI diagnosis [127,128,129,130]. MRI is also capable of detecting non-hemorrhagic lesions related to DAI, such as widespread disruption of axons and local edema associated to axonal damage [10], with MRI sequences like diffusion tensor imaging (DTI), diffusion weighted imaging (DWI), MR spectroscopy, and conventional MR sequences [131,132,133].

**Figure 3:**
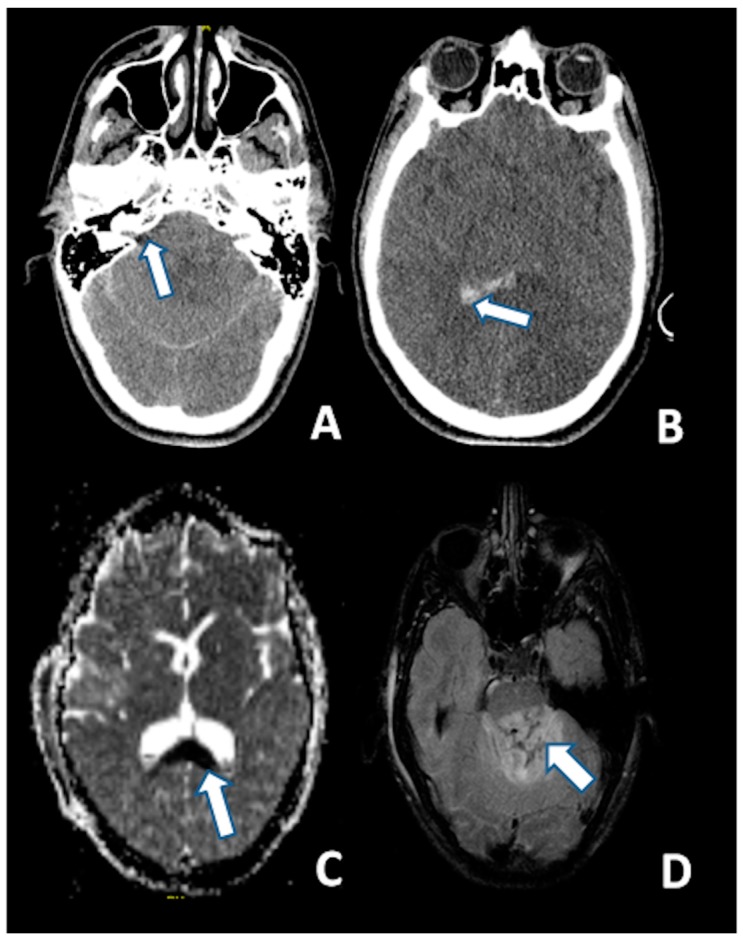
CT at 6 h after the trauma: (**A**) Absence of the basal cisterns (arrow); and (**B**) massive brain edema and intraventricular hemorrhage (arrow); MRI scan of the same patient 5 days after the trauma: (**C**) DWI sequence disclosing the axonal injury of the corpus callosum (arrow); and (**D**) injury of the dorsal aspect of the pons (arrow) (FLAIR).

T2-weighted and, particularly FLAIR sequences, provide good visualization of non-hemorrhagic parenchymal lesions, but DWI has a greater sensitivity for identifying DAI as hyperintense lesions (Figure 3) [134].

Diffusion tensor magnetic resonance imaging (DTI) represents a promising method for the non-invasive detection of the degree of fiber damage. A recent study [134] evaluated diffusion tensor tractography (DTT) as an instrument for identifying diffuse axonal injury in nineteen patients with acute, mild, and moderate traumatic brain injury (TBI), using fractional anisotropy (FA) that represents the degree of alignment of the white matter tracts [135], and mean diffusivity (MD), which represents the degree of overall restrictions to water diffusion, as a measure of the average diffusion in all directions [136]. The reduction in FA and changes in MD were significant, compared with a control group, in several tracts such as the corpus callosum, fornix, uncinate fasciculus, superior and posterior thalamic radiations, and inferior longitudinal fasciculus. Furthermore, they observed a meaningful correlation between FA and MD indices and the seriousness of post-concussive symptoms, indicating their possible utility as predictors of long-term outcome.

In clinical practice, 1.5 T MRI seems to be sufficient for the detection of DAI-related micro-hemorrhagic lesions. Nevertheless, T2*-weighted gradient-echo MRI at 3 T is found to be superior to MRI at 1.5 T for this purpose. Therefore, it could be appropriate when there is a strong clinical suspicion of DAI without evident lesions in routine MRI, and when a long interval since the trauma has passed [126].

In spite of this, patients with closed-head trauma suffering from post-traumatic cognitive impairment may not present pathological findings at 1.5 or 3 T MRI [137]; MRI at higher field strength with a better spatial resolution, such as 7 T MRI, is more sensitive in visualizing microhemorrhagic DAI. The role of 7 T SWI has been investigated by Moenninghoff et al. [10] who stated its importance as a tool for diagnostic in inconclusive or medicolegal cases.

Residues of microhemorrhage may last for months or years, though hemorrhagic DAI lesions become less evident after three months [138,139].

Moen et al. [138] examined the evolution of TAI lesions during the first year post-injury using conventional MRI in the early phase and at three and 12 months, demonstrated attenuation of volume and number of non-hemorrhagic TAI lesions on FLAIR sequences during the first three months after TBI. Brainstem lesions were often no longer visible, leading to a lower stage of TAI—this is an important finding since brainstem lesions have been associated with poor outcomes [140].

In terms of long-term follow-up imaging, it should be considered that DAI may result in atrophy [141]; moreover, in the chronic stages of a TBI white matter hyperintensities in FLAIR sequences could be ascribed to post-traumatic gliosis [142].

Summary: CT scan is the golden standard for a first emergency level of examination. MRI performed with contemporary sequences and methodology allows a more accurate definition of the damage on the brain parenchyma.

## 7. Conclusions: What Is Currently Ascertained

DAI is caused by a blunt concussive trauma which applies strong rotational and translational forces to the brain parenchyma, resulting in a diffuse disruption of the white matter axons. TBI results in the delayed dysfunction and death of neuronal population near and distant to the site of injury. This secondary phase of increased neuronal vulnerability contributes to many of the neurological conditions associated with traumatic injury. DAI is not only caused by primary axotomy from mechanical forces, but also from secondary axotomy due to a progressive molecular and cellular cascade of pathologic changes within the axon after initial shear stress at the time of injury. Studies support the role of an altered axonal calcium homeostasis in the mechanism of secondary damage of axon, and suggest that calcium channel blockers that can alleviate the secondary damage, as well as other mechanisms implied in the secondary injury, could be targeted as candidates of therapeutic approaches. ROS-mediated axonal degeneration is mainly caused by extracellular Ca^2+^. Removal of extracellular Ca^2+^, rather than a blockade of mitochondrial Ca^2+^ release, is an efficient strategy in lowering intracellular Ca^2+^ and inhibiting spheroid formation. In vivo, calpain and calcineurin inhibition is able to mitigate axonal degeneration. Oxidative stress with resulting damage of the endogenous antioxidant defense mechanisms acts as a significant player in the secondary events leading to neuronal death. Increase in the defense mechanisms through the use of exogenous antioxidants may be neuroprotective, particularly if they are given within the neuroprotective time window [143,144]. A promising potential therapeutic target in DAI is to directly address mitochondria related injury or to modulate energetic axonal energy failure [3]. mPTP is produced as a consequence of a greater gathering of calcium and permits the movement of small molecules inside or outside the mitochondria. mPTP participation in the axonal degeneration process has led to considering it as a therapeutic goal in DAI. However, dietary supplementation with antioxidant and the use of pharmacological agents targeting oxidative stress seem logical but the benefits of proven antioxidant strategies have not been clearly demonstrated to date [145].

## Figures and Tables

**Table 1:** Potential biomarkers of DAI.

Precursor	Proteolytic System	Subunits	Potential Biomarkers
αII spectrin	Caspase-3 and calpain	Spectrin breakdown products (SBDP)	SBDP145, SBDP150, SBDP120
Neurofilament protein (NF)	Caspase and calpain	Light (NFL), medium (NFM), and heavy (NFH) neurofilament subunit protein	pNF-H
Glial fibrillary acidic protein (GFAP)	Calpain	GFAP breakdown products (GFAP-BDP)	GFAP-BDP38, GFAP-BDP44
Microtubule-associated protein tau (MAP-tau)	Caspase-3 and calpain	Tau breakdown products (TauBDP)	TauBDP45 TauBDP35
β-amyloid precursor protein (β-APP)	Caspase-3	Amyloid beta peptides	Amyloid β peptide42 (Aβ42)
